# A Consolidated Framework for the Detection of Alzheimer’s Disease Using EEG Signals and Hybrid Models

**DOI:** 10.3390/biomimetics11050348

**Published:** 2026-05-15

**Authors:** Sunil Kumar Prabhakar, Dong-Ok Won

**Affiliations:** 1 Department of Artificial Intelligence Convergence, College of Information Science, Hallym University, Chuncheon 24252, Republic of Korea; sunilprabhakar22@hallym.ac.kr; 2 College of Medicine, Hallym University, Chuncheon 24252, Republic of Korea; 3 Department of Population and Quantitative Health Sciences, University of Massachusetts Chan Medical School, Worcester, MA 01655, USA

**Keywords:** feature extraction, feature selection, Alzheimer’s disease, EEG, classification

## Abstract

Alzheimer’s disease (AD) is a serious neurodegenerative disorder that can severely affect behavior and thinking patterns, and is accompanied by frequent memory loss. The early diagnosis of AD is essential, as this can benefit the patient, but detecting AD is a complex process due to the nature of its associated clinical data. Electroencephalography (EEG) serves as a promising and cost-effective technique for analyzing AD-related brain activity patterns. In this work, a consolidated framework for detecting AD using EEG signals and hybrid models is proposed that uses a dataset that is available online. For the feature extraction module, five efficient techniques—Principal Component Analysis (PCA), Kernel Partial Least Squares (KPLS), Kriging Model, Isomap, and K-means clustering—are used. For feature selection, with the help of biomimetics-based concepts, three efficient algorithms are used: hybrid Cuckoo Search Optimization–Rat Swarm Optimization (CSO-RSO), Zebra Optimization (ZOA), and hybrid Gravitational Search Algorithm–Particle Swarm Optimization (GSA-PSO). Four interesting hybrid classifiers are utilized here to detect AD using EEG signals—hybrid Extreme Learning Machine–Adaboost (ELM–Adaboost), hybrid Classification and Regression Trees–Adaboost (CART–Adaboost), and hybrid weighted broad learning system-based Adaboost (HWBLSA), followed by a hybrid machine learning classification model with a soft voting technique—and, finally, these are compared with other standard machine learning classifiers. The highest classification accuracy of 98.71% is found when the Kriging Model feature extraction concept is combined with the hybrid GSA-PSO feature selection method and classified with the ELM–Adaboost classifier.

## 1. Introduction

AD, a prominent neurological disorder of the brain, gradually causes degeneration of neuronal cells [1]. Its origins remain unclear, but patients suffering from AD experience symptoms of cognitive decline, memory loss, and sometimes hallucinations. AD is often a progressive disease that develops slowly with few symptoms that do not affect the daily lives of patients, but later can lead to severe deterioration, affecting the patient and their family to a great extent [2]. In the initial stages of AD, its phenotype follows a pattern of Mild Cognitive Impairment (MCI) and is identified by memory loss. Early diagnosis is important so that the condition can be treated and managed efficiently and—to a certain extent—successfully; however, there is no cure for AD [3]. Distinguishing AD symptoms from signs of normal aging is quite difficult; brain tissue is usually examined to diagnose the condition. However, non-invasive techniques are still being investigated that may enable precise and definitive AS diagnoses, and the medical community is hopeful that successful results will be achieved soon [4]. With the aid of various brain imaging techniques alongside psychological tests, AD can be diagnosed, but this still depends on multiple factors including the skill of the neurologist [5]. Understanding and interpreting the correlation between biomarkers in diagnostic tests is still very difficult, and sometimes time-consuming and expensive. Thus, EEG serves as a wonderful tool as it is widely available, easy to use and maintain, and inexpensive; for these reasons, it is widely preferred in the research community [6].

Due to the physiological activity of neurons, electrical potential is generated, allowing this activity to be recorded using EEG [7]. Cell membranes can be depolarized easily, which helps to generate electric currents, creating waves that can be detected using scalp electrodes. The activity of a single neuron cannot be recorded, but when a group of neurons acts synchronously, it can be captured as an EEG signal [8]. A trained neurologist inspects the EEG signals visually, but this is a time-consuming task as it involves a lot of noise and artifacts. Multiple neural functions exhibit non-linear dynamics, and so more sophisticated techniques must be utilized to assess the behavior of such signals [9]. Computational analysis of EEG signals has been very successful, and various techniques have shown promising results in the analysis and classification of brain disorders. Various techniques such as time–frequency analysis, information theory analysis, and graph theory analysis are used to analyze and distinguish healthy and unhealthy subjects [10]. The EEG rhythm of people diagnosed with AD shows a slow pattern, and the complexity of EEG signals can vary greatly. The intention of this study is to apply a framework with efficient feature extraction and selection and hybrid classification models to successfully detect AD [11]. Before presenting the proposed models, the most prominent studies performed in this field for the classification of AD are discussed in the following.

The classification of AD patients with the help of EEG signal processing was reviewed by Fiscon et al. [12]. A comprehensive review on resting-state EEG for diagnosis and progressive assessment of AD was completed by Cassani et al. [13]. EEG signal processing techniques were combined with supervised techniques and discrete Fourier and wavelet transforms for Alzheimer’s patient classification by Fiscon et al., who reported a high accuracy of 92% [14]. EEG modulation spectral patch features were used to diagnose and detect the severity of AD by Cassani and Falk, who obtained an accuracy of 88% [15]. Bi and Wang employed EEG spectral images along with deep learning to analyze early AD diagnosis, where spectral topography maps were analyzed with a spike convolutional deep Boltzmann machine, reporting an accuracy of 95.04% [16]. With reference to physiological aging, Vecchio et al. used many innovative EEG biomarkers along with a machine learning model to classify AD, for which an accuracy of 95% was obtained [17]. Ieracitano et al. employed a novel multimodal machine learning concept using continuous wavelet transform (CWT) with bispectrum features and a Multi-Layer Perceptron (MLP) classifier, yielding a classification accuracy of 89.22% [18]. Conventional machine learning and recurrent neural network (RNN) models were used by Seo et al. for the full classification of AD patients, obtaining an average accuracy of 70.97% [19]. A resting-state EEG signal utilizing CWT tiled topographical images and AlexNet-based Convolutional Neural Networks (CNNs) were used by Huggins et al., who reported an accuracy of 98.9% [20]. For the automated diagnosis of brain disorders like AD and schizophrenia, Alves et al. used the concept of EEG functional connectivity and deep learning, with 100% accuracy [21]. Pirrone et al. described a supervised machine learning concept utilizing power spectrum density, short-time Fourier transform, and K-nearest neighbors (KNNs) for AD detection, reporting a classification accuracy of 86% [22]. The concept of EMD with Hjorth parameters, Kruskal–Wallis analysis, and SVM was analyzed by Puri et al., who reported an accuracy of 92.9% [23]. For the early diagnosis of AD, Rossini et al. developed integrated biomarkers along with machine learning techniques, implementing graph theory with SVM, and reported a classification accuracy of 95% [24].

Dogan et al. employed primate brain pattern-based automated detection of AD using EEG signals, with 100% accuracy reported [25]. A graph neural network (GNN) approach with functional connectivity nodes was employed by Klepl et al. for AD classification analysis, with an accuracy of 84.7% [26]. Some efficient computational techniques for analyzing EEG signals with respect to AD classification were discussed in detail by Vicchietti et al. [27]. Resting-state EEG was used by Zheng et al. to diagnose AD by integrating complexity, spectrum, and synchronization signal features, and a high classification accuracy of 95.86% was reported [28]. Detailed EEG-dependent classification of the stages of AD and MCI was achieved by Calub et al. [29]. With the aid of low-complexity orthogonal wavelet filter banks and SVM, the automatic detection of AD from EEG signals with a classification accuracy of 98.6% was reported by Puri et al. [30]. Sen et al. classified AD using EEG signals and deep learning, in which the concept of proper rotation components was extracted from the EEG signals and then implemented with 1D-CNN. An accuracy of 94% was obtained [31]. The resting-state EEG microstate features for AD classification were obtained with conventional machine learning classifiers, with a high accuracy of 99.22% reported by Yang et al. [32]. To quantify communication between electrode pairs for the efficient classification of AD and frontotemporal dementia, Ma et al. used Support Vector Machines (SVMs), reporting a high accuracy of 96.6% [33]. A comprehensive analysis of the discriminative features of EEG-based classification of AD and frontotemporal dementia was performed by Rostamikia et al. [34]. A Lattice 123 pattern for automated AD using EEG signals was employed by Dogan et al., who reported a classification accuracy of more than 98% [35]. Two channel EEG features were analyzed by Jang et al. for dementia classification with an Extreme Gradient Boosting model, reporting a balanced accuracy of 97.05% [36]. EEG signals and a few images of clock drawing tests were used with ensemble learning for AD classification, and this interesting strategy was proposed by Huh et al. [37]. The concept of dual attention and Optuna-optimized SVM with deep learning methods was presented by Arikan et al. for AD classification using EEG data [38]. The concept of synchrosqueezing transform and deep transfer learning was used for AD detection by Jain and Srivastava, who reported a high classification accuracy of 98.5% [39]. The concept of EEG phase synchronization was used by Cao et al. for AD analysis, in which a brain network analysis was constructed and a graph convolutional network was used with an average classification accuracy of 77.8% [40]. A multiscale temporal deep network was used for AD classifiers for EEG by Zini et al. [41], and in another study utilizing PSO, dimensionality reduction and conventional machine learning classifiers were used by Lopez and Varas, where a classification accuracy of over 95% was obtained [42]. Additionally, hippocampal microstructural signatures that unveil the stage-specific pathways in Alzheimer’s disease progression were discussed by Yu et al. in [43], and the efficacy of brain power mapping concepts with optimized deep learning for analyzing EEG data was discussed in detail by Chen et al. in [44]. In this work, the following workflow is proposed. Once the basic pre-processing step is completed using Independent Component Analysis (ICA), the work is conducted as follows.

(i) For the feature extraction module, five efficient techniques like Principal Component Analysis (PCA), Kernel Partial Least Squares (KPLS), Kriging Model, Isomap, and K-means clustering techniques are used.
(ii) For feature selection, three efficient algorithms are used: hybrid Cuckoo Search Optimization–Rat Swarm Optimization (CSO-RSO), Zebra Optimization (ZOA), and hybrid Gravitational Search Algorithm–Particle Swarm Optimization (GSA-PSO).
(iii) Four interesting hybrid classifiers are utilized here to detect AD using EEG signals: hybrid Extreme Learning Machine–Adaboost (ELM–Adaboost), hybrid Classification and Regression Trees–Adaboost (CART–Adaboost), and hybrid weighted broad learning system-based Adaboost (HWBLSA), followed by a hybrid machine learning classification model with soft voting technique—and finally, these are compared with other standard machine learning classifiers.

**Figure 1:**
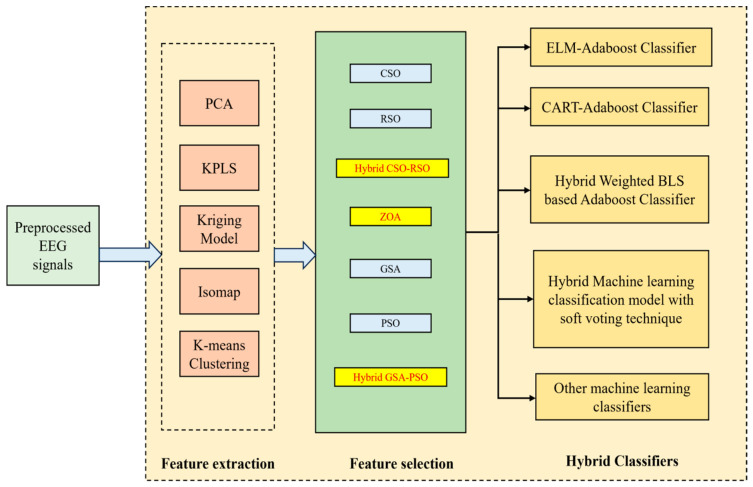
Simplified illustration of the overall workflow.

Figure 1 is a simplified schematic representation of the entire workflow employed in this research.

This manuscript’s structure is organized as follows: Section 2 discusses the feature extraction techniques and Section 3 discusses the feature selection techniques employed in this study. The classifiers used in this work are discussed in Section 4 and the results and a discussion of them are provided in Section 5. This paper is concluded in Section 6.

## 2. Feature Extraction Techniques Utilized in This Work

The feature extraction schemes PCA, KPLS, Kriging Model, Isomap, and K-means Clustering are explained in this section.

### 2.1. PCA

For achieving optimal performance, effective features must be chosen, and PCA aids greatly in this process [45]. In the field of multivariate statistics, it is a popular method and the most famously used unsupervised technique for choosing features. The dimensionality of the data is mitigated by PCA so that only the important attributes remain in the data. The total number of variables can be reduced with the utility of orthogonal pairings used with a wide range of dissimilarity. The important subset of the dataset can be chosen by the PCA so that it can be categorized clearly and easily. The main idea of PCA lies in the projection principle. The original data $P\in Z^{m}$ are present where m is the number of columns and can be predicted into a specific subspace with fewer size elements, represented as $P\in Z^{k}$, and the data integrity of it is maintained easily. The implementation of PCA is as follows:

From a set of m dimensions to a set of k dimensions, the feature dimensionality is mitigated using pre-processing and dimensionality reduction. The mean and variance of the data are standardized during pre-processing, and in the second phase, the construction of the covariance matrix and eigenvectors is framed. Based on the following equation, the mean and standard deviation are computed so that the input features are standardized:

(1) $$\mu =\frac{1}{n}\sum_{t=1}^{n}P_{\left(i\right)}$$

where the number of cases is represented as n and the datapoints are represented as $P_{\left(i\right)}$. The parameter $P_{\left(i\right)}$ is substituted with $P_{\left(i\right)}-\mu$. Unit variance is obtained so that each vector $P_{h(i)}$ is transformed as follows:

(2) $$\sigma_{i}^{2}=\frac{1}{n}\Sigma_{i}{\left(P_{h(i)}\right)}^{2}$$

Every $P_{h(i)}$ is replaced with $\frac{P_{h(i)}}{\sigma }$. The covariance matrix $Cov_{n}$ is now calculated as follows:

(3) $$Cov_{n}=\frac{1}{n}{\sum \left(\left(P_{\left(1\right)}\right)P_{\left(i\right)}\right)}^{T}$$

The eigenvalues and eigenvectors of $Cov_{n}$ are computed successfully. The eigenvalues are diminished by managing and setting the eigenvectors. The h eigenvectors are chosen and the highest eigenvalues obtained are used to produce the components C. Using C and the following equation, the data are converted to the new subspace as follows:

(4) P=C×Q

where P represents a 1×e vector representing a sample and p represents the converted k×1 sample in the novel subspace. The number of attributes contributes a lot to the performance and computation of PCA, as it specifies the datapoints clearly.

### 2.2. KPLS

When the conventional PLS is improved, it then becomes KPLS [46]. In a high-dimensional space, the input variables present in the latent information can be extracted successfully by introducing the Kernel. It is presumed that the training dataset comprises both input and output variable matrices represented as $P\in {ℜ}^{n\times m}$ and $Q\in {ℜ}^{n\times 1}$, where the sample dimension is indicated as m and the sample size is indicated as n. The mapping function is indicated as $\phi \left(p\right)$, where the training dataset is mapped to a high-dimensional space from the original space. The mapped vectors corresponding to $p_{i}$ and $p_{j}$ are indicated by $\phi_{i}$ and $\phi_{j}$, respectively. The expression of Kernel function is as follows:

(5) $$K\left(p_{i},p_{j}\right)=\phi_{i}\phi_{j}^{T}$$

where the Gaussian Kernel function is indicated as $K\left(\cdot \right)$ and is expressed as follows:

(6) $$K\left(p_{i},p_{j}\right)=\exp\left(-\gamma {\left\|p_{i}-p_{j}\right\|}^{2}\right)$$

where the Kernel parameter is specified by γ. The input variable matrix can be converted into a Gram matrix with the help of the above transformation and is expressed as follows:

(7) $$K=\left[\begin{array}{ccc} \phi_{1}^{T}\phi_{1} & \ldots & \phi_{1}^{T}\phi_{n}\\ \vdots & \ldots & \vdots \\ \phi_{1}^{T}\phi_{1} & \ldots & \phi_{1}^{T}\phi_{n} \end{array}\right]=\left[\begin{array}{ccc} K\left(p_{1},p_{1}\right) & \ldots & K\left(p_{1},p_{n}\right)\\ \vdots & \ldots & \vdots \\ K\left(p_{n},p_{1}\right) & \ldots & K\left(p_{n},p_{n}\right) \end{array}\right]$$

The decomposition of the training data based on the non-linear PLS technique is expressed as follows:

(8) $$K=YA^{T}+C$$

(9) $$Q=ZB^{T}+D$$

For the input variable matrix, the loading matrix is specified as A and for the output variable matrix, the loading matrix is specified as B. Residual matrices are represented as C and D, respectively. A significant specification of Y and Z is expressed as follows:

(10) $$Y=\left[y_{1},y_{2},\ldots ,t_{b}\right]$$

(11) $$Z=\left[z_{1},z_{2},\ldots ,z_{b}\right]$$

where the total number of latent variables is represented as b. K and Q are scaled to a zero-mean level so that the decomposition becomes easier and is specified as follows:

(12) $$K\leftarrow \left(I_{n}-\frac{1}{n}1_{n}1_{n}^{Y}\right)K\left(I_{n}-\frac{1}{n}1_{n}1_{n}^{Y}\right)$$

(13) $$K_{y}\leftarrow \left(K_{y}-\frac{1}{n}1_{ny}1_{n}^{Y}K\right)\left(I_{n}-\frac{1}{n}1_{n}1_{n}^{Y}\right)$$

where an identity matrix is specified as $I_{n}$ and it has n-dimensionality. A vector with length n and value 1 is specified by $1_{n}$ and a vector with length ny and value 1 is specified by $1_{ny}$. For the input variable matrix of both test data and training data, the respective Kernel latent matrix is specified as follows:

(14) $$Y=KZ{\left(Y^{Y}KZ\right)}^{-1}$$

(15) $$Y_{y}=K_{y}Z{\left(Y^{Y}KZ\right)}^{-1}$$

### 2.3. Kriging Model

The random process models can be constructed using an interpolation technique called the Kriging Model [47]. The values of the data correlations are fully analyzed and a spatial correlation is exhibited between the neighboring points so that the variable values can be projected well. A lot of unbiased estimates can be provided by the Kriging Model for the unknown information, as follows:

(16) $$h^{*}\left(p_{0}\right)=\sum_{i=1}^{N}\lambda_{i}h\left(p_{i}\right)$$

where the estimated values are represented as $h^{*}\left(p_{0}\right)$. The observed value is represented as $h\left(p_{i}\right)$ and its specific weights are expressed as $\lambda_{i}$ at location $p_{i}(i=1,\ldots ,N)$, where the number of sampling points is denoted by N and it helps to assess and estimate the total number of measurement points in the technique. Mostly, a semi-variogram function $\gamma \left(p,d\right)$ is introduced by the Kriging technique so that the correlation can be characterized accurately. The weights can be computed so that the spatial characteristics of variables can be identified sufficiently by the Kriging method, as follows:

(17) $$2\gamma =E\left\{{\left[H\left(p\right)-H\left(p+d\right)\right]}^{2}\right\}-\left\{E{\left[H\left(p\right)-E\left[H\left(p+d\right)\right]\right]}^{2}\right\}$$

where the two points in space are identified as p and p+d. The distance between them is represented as d, and the expectation operator is denoted as E. At a location p, the values of the random variable are specified by $H\left(p\right)$.

### 2.4. Isomap

The concept of classical scaling is quite successful in multiple applications but as it must always retain Euclidean distances, it can be a drawback in some applications. The classical scaling concept does not address the neighboring datapoints and so it finds it difficult to deal with datapoints present near the manifold whose size is much greater than the interpoint distance. To overcome this drawback, Isomap was proposed as it is a versatile technique enabling the curvilinear distance present in between the datapoints to be preserved [48]. Over the manifold, the measurement between two points is analyzed as the distance of curvilinear nature. Under the Isomap concept, the curvilinear distances for datapoint $p_{i}(i=1,2,\ldots ,n)$ are calculated by means of assembling a neighboring graph G so that each datapoint $p_{i}$ can be connected easily with its respective k nearest neighbor $p_{ij}\left(j=1,2,\ldots ,k\right)$ in the dataset P. Between these two points, the curvilinear distance can be estimated and is represented as the shortest distance between the two points in the entire graph; it is established with the help of Dijkstra’s algorithm. Between all the datapoints in P, the curvilinear distance is calculated so that a pairwise curvilinear distance matrix is found. The concept of classical scaling is implemented and the low-dimensional specifications $q_{i}$ of the datapoints $p_{i}$ are computed on the obtained curvilinear distance matrix. Though Isomap can sometimes suffer from topological instability, it can still be managed well by adjusting the parameters of Dijkstra’s algorithm slightly.

### 2.5. K-Means Clustering

The samples with similar properties are collected separately and the samples with dissimilar properties are collected separately with the aid of clustering techniques. In the implementation of data analysis schemes, the K-means clustering algorithm is quite popular as it is easily implemented. K-means clustering is also highly scalable and flexible and so it is applied to multiple objective optimization schemes [49]. In different ways, the centroid of the K-means clustering can be defined easily, as follows:

(18) $$C_{h}\left(h=1,2,\ldots ,k\right)$$

so that the mean value can be assigned precisely to the different objects in the cluster. The Euclidean distance helps to assess the distance between the specifications of the cluster $s_{h}$ and the object $O_{b}\in C_{h}$ and it is represented as follows:

(19) $$dis\left(O_{b},s_{h}\right)$$

The distance between all the datapoints and its respective cluster centers is considered and validated using the clustering quality parameter. The sum of the distance $\left(S_{D}\right)$ can be mathematically expressed as follows:

(20) $$S_{D}=\sum_{i=1}^{k}\sum_{O_{b}\in C_{h}}dist\left(O_{b},s_{h}\right)$$

(21) $$s_{h}=\frac{1}{n_{h}}\sum_{O_{b}\in C_{h}}O_{b}$$

where the number of clusters is denoted by k. In the $h^{th}$ cluster, the total number of datapoints is indicated by $n_{h}$. The total number of clusters $\left(k\right)$ in the K-means clustering technique must be manually specified as it is quite sensitive to the cluster center process initially. The sensitivity measures of the K-means clustering process can be improved greatly but this involves a highly computational procedure. The K-means clustering technique has good global search capabilities and so it can also be implemented for many multi-objective optimization problems.

## 3. Feature Selection Techniques Utilized in This Work

Biomimetics is an interdisciplinary field that analyzes the time-tested patterns of nature and helps to develop sustainable strategies and innovative solutions to various engineering and mathematical problems [50]. Biological structures and processes are studied thoroughly so that sustainable and highly optimized designs can be developed and implemented successfully in this field. Nature, in a biomimetics context, can be considered a model, mentor, or efficient measure to judge the sustainability of innovations. Biomimetics applications can be implemented in other fields like the natural sciences, architecture, medicine, and robotics [50]. The main feature selection techniques utilized in this work are the hybrid CSO-RSO, ZOA, and hybrid GSA-PSO algorithm.

### 3.1. Hybrid CSO with RSO Algorithm

#### 3.1.1. CSO

One of the famous optimization algorithms used is CSO and it is highly influenced by the attitude of brood parasitism present in the environment [51]. Cuckoo birds do not have the habit of constructing independent nests as they generally bury their eggs in other nests and nurture them carefully. To trace the nest position, levy flights are utilized instead of random walks. Once the egg is determined by the host as not its own egg, then it can discard the egg based on the situation. The expression of Levy flight is given as follows:

(22) $$z_{k}^{\left(t+1\right)}=z_{k}^{\left(t\right)}+\alpha ⊕Levy\left(l,\lambda \right)$$

The step length is indicated by α and the f Levy circulation is specified as follows:

(23) $$Levy\left(l,\lambda \right)\sim l^{-\lambda },\left(1<\lambda \leq 3\right)$$

The step length is indicated by l, and the above Equation (23) signifies the mean and its corresponding variance. If the nest is good and has a large majority of support from the birds, it progresses towards the next step. A hybrid of local and global random walks is employed by this algorithm and it can be assessed by the switching parameters.

(24) $$w_{k}^{t+1}=w_{k}^{t}+\gamma \cdot L\cdot H\left(p_{a}-\varepsilon \right).\left(w_{k}^{t}-w_{m}^{t}\right)$$

where the new progression is expressed as $w_{k}^{t+1}$ and the earlier progression is expressed as $w_{k}^{t}$. The chosen solutions are expressed as $w_{k}^{t}$ and $w_{m}^{t}$. The Heaviside side is specified by $H\left(\cdot \right)$ and the random number is expressed by ε. For each cuckoo, Levy flight is used to assess the random walk, as follows:

(25) $$w_{k}^{t+1}=w_{k}^{t}+\gamma \cdot M_{\alpha }(L)$$

The $M_{\alpha }(L)$ specifies the random walk length and is often projected as a Levy circulation, which has a variance and mean for each k, and is represented as follows:

(26) $$M_{\alpha }(L)∼\frac{1}{L^{\alpha +1}};\left|L\right|\gg 1$$

where the probability of obtaining the Levy random number is specified by α.

#### 3.1.2. RSO

Inspired by the behavior of rats, this algorithm was developed and is widely used [52]. Rats display multiple movements such as jumping, chasing, and tumbling, and at times, rats can become aggressive in certain environments based on various triggering factors. To enhance the speed and local search ability of the algorithm, RSO is hybridized with CSO so that the optimal feature subset can be assessed accurately.

#### 3.1.3. Implementation of the CSO-RSO Algorithm

The simplified implementation of the CSO-RSO algorithm is explained as follows.

Step 1: Start process:

The population of the rats $r_{k}$ is initialized and then, the population of host nests of cuckoo search $z_{k}$ is also initialized, where k=1,2,…,n and is represented as follows:

(27) $$R=Q\cdot R_{k}(z)+W\cdot \left(R_{s}\left(z\right)-R_{k}\left(z\right)\right)$$

where the rat’s position is assigned as $R_{k}(z)$. Q is a parameter generated to assign the random numbers in the process.

Step 2: Generation of random numbers:

Once the initialization process is completed, random features are generated with the help of this hybrid algorithm, as follows:

(28) $$Q=A-z\times \left(\frac{A}{Max_{Iter}}\right)$$

where $z=0,1,2,\ldots ,Max_{Iter}$.

(29) W=2⋅rand( )

where A is a random number assigned in the range of [0, 1]. Q is responsible for controlling the total number of iterations.

Step 3: Fitness function evaluation:

For each search agent, the fitness value is estimated. If $r_{k}$ is identified as a good search agent, then $\left(z<Max_{Iter}\right)$ is satisfied. The exploration of a good search agent is completed here.

Step 4: Update of position of search agent:

With the help of Equation (30), the search agent position of the rat is updated as follows:

(30) $$R_{k}(z+1)=\left|R_{a}(z)-R\right|$$

where the updated rat location is expressed by $R_{k}(z+1)$.

Step 5: Optimal feature subset determination:

The search agent fitness value is computed and updated, and finally, $R_{a}$ is also updated. Using Equations (26) and (30), the best feature subset is chosen.

**Figure 2:**
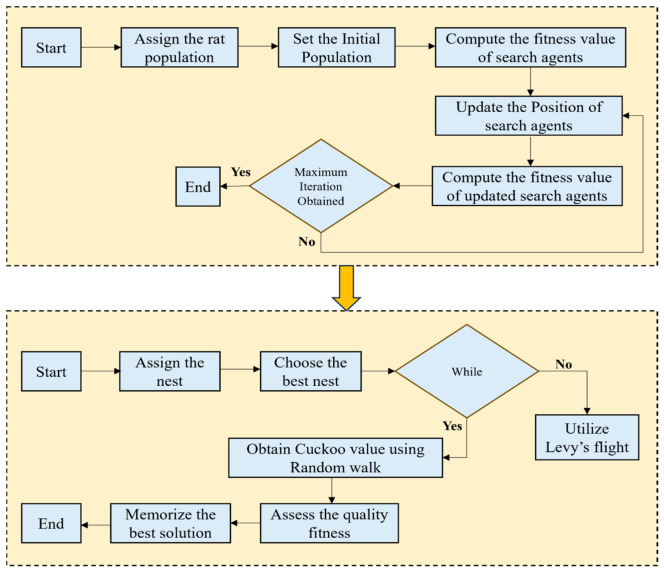
Overall model of the hybrid CSO-RSO algorithm.

Step 6: End of process:

Once the conditions are met, the process is terminated; otherwise, it is repeated until the criteria are met. Therefore, the output of the hybrid CSO-RSO will assess the optimal feature set which is to be fed to the classifiers. Figure 2 shows the overall model of the hybrid CSO-RSO algorithm.

### 3.2. ZOA

This intelligent optimization algorithm was inspired by the movement exhibited by zebras in nature [53]. Zebras generally live in communities, enjoying the company of each other. However, they exhibit two important survival strategies: the foraging technique and the defense technique. Based on these two survival mechanisms, the ZOA was developed. The implementation of this algorithm can be explained as follows:

Depending on the population concept, ZOA was developed as it comprises many zebra individuals. For representing the solution space, the ideal candidate solutions are identified by these zebra individuals. The values of the related decision variables help to assess the search space position. Every zebra is assessed as a mathematical vector and with the help of a matrix, it can be visually expressed as follows. The population matrix of ZOA is expressed as follows:

(31) $$Y={\left[\begin{array}{c} Y_{1}\\ Y_{j}\\ \vdots \\ Y_{N} \end{array}\right]}_{N\times d}={\left[\begin{array}{cccc} y_{1,1} & y_{1,k} & \ldots & y_{1,d}\\ y_{j,1} & y_{j,k} & \ldots & y_{j,d}\\ \vdots & \vdots & \vdots & \vdots \\ y_{N,1} & y_{N,k} & \ldots & y_{N,d} \end{array}\right]}_{N\times d}$$

where the specification of the zebra population is identified by Y. The $j^{th}$ zebra is specified as $Y_{j}$ and the $j^{th}$-dimensional decision mode of the $j^{th}$ zebra is indicated as $y_{j,k}$. In the population, the members are represented as N and the total number of decision nodes is represented as d. To any problem, a potential solution is specified by every zebra. Once the decision variable value is accessed, its fitness value is computed. A vector is obtained if it is substituted into the fitness function and is expressed as follows:

(32) $$F=\left[\begin{array}{c} F_{1}\\ F_{j}\\ \vdots \\ F_{N} \end{array}\right]={\left[\begin{array}{c} F\left(Y_{1}\right)\\ F\left(Y_{j}\right)\\ \vdots \\ F\left(Y_{N}\right) \end{array}\right]}_{N\times 1}$$

where the fitness value vector is specified by F. For the $j^{th}$ zebra, the fitness value is indicated by $F_{j}$. For each member, the fitness value is evaluated and the candidate solutions are analyzed. Finally, the perfect candidate solutions are accurately identified.

Phase 1: Foraging Mode

Grass is a staple food for zebras, some of which have peculiar foraging habits. The plain zebra is represented as the pioneer zebra in this work, and they exhibit a poor level of nutrition. The ecological niche is promoted by this behavior in zebras and so a novel foraging space is given to other species that can gnaw on the lower layer of grass, and this group exhibits a high level of nutrition. During the foraging stage, the zebra positions are modeled mathematically as follows:

(33) $$y_{\left(k,j\right)}^{\left(new,P_{1}\right)}=y_{\left(k,j\right)}+r\cdot \left(PZ_{k}-J\cdot y_{j,k}\right)$$

(34) $$Y_{i}=\left\{\begin{array}{l} Y_{i}^{new,P_{1}},F_{i}^{new,P_{1}}<F_{i}\\ Y_{i},\;else \end{array}\right.$$

Before updating the $j^{th}$ zebra, the value of the $k^{th}$-dimension decision mode can be expressed as $y_{j,k}$.

After updating the $k^{th}$ dimension for the $j^{th}$ zebra, the updated value is represented as $y_{j,k}^{new,P_{1}}$. The random number is represented as r and lies in the range of [0, 1]. The pioneer zebra is specified as PZ and the value of its $k^{th}$ dimension is represented as $PZ_{k}$. J=round(1+rand), where rand represents a random number in the range of [0, 1] and so $J\in \left\{1,2\right\}$. Depending on the first stage, the updated position of the $j^{th}$ zebra is specified as $Y_{j}^{new,P_{1}}$. Before updating the $j^{th}$ zebra, the corresponding fitness value is represented as $F_{i}$. The novel fitness value with respect to the novel position of the $j^{th}$ zebra is represented as $F_{i}^{new,P_{1}}$. If the obtained fitness value is high, then the updated one replaces the original position.

Phase 2: Defense Mode

The threat of predators must be efficiently dealt with by the zebras as they face a high threat from lions, cheetahs, hyenas, tigers, wolves, and wild dogs. The zebras utilize the escape mode when facing target predators like lions and tigers and are mathematically expressed as follows:

(35) $$y_{\left(j,k\right)}^{\left(new,P_{2}\right)}=\left\{S_{1}:y_{j,k}+C\cdot \left(2c-1\right)\cdot \left(1-\frac{t}{T}\right)\right.\cdot y_{j,k},P_{s}\leq 0.5$$

(36) $$y_{\left(j,k\right)}^{\left(new,P_{2}\right)}=\left\{S_{2}:y_{j,k}+r.\left(RZ_{k}-J\cdot y_{j,k}\right)\right.,else$$

When faced with the threat of small-sized predators like wild dogs, zebras tend to initiate a system so that they can scare them off easily, and this is represented as follows:

(37) $$Y_{j}=\left\{\begin{array}{l} Y_{j}^{new,P_{2}},F_{i}^{new,P_{2}}<F_{i}\\ Y_{j},\;else \end{array}\right.$$

where the $k^{th}$-dimension decision node of the $j^{th}$ zebra is represented as $y_{j,k}$ before being updated. The updated value of the $k^{th}$-dimension decision node of the $j^{th}$ zebra is represented as $y_{j,k}^{new,P_{2}}$. Constant is represented as C and is set as 0.01 in our experiment and the random number is represented by r and is in the range of [0, 1]. The current iteration is represented by t and maximum iteration is specified as T. RZ specifies the randomly chosen individual zebra and $RZ_{k}$ expresses the value of its $k^{th}$ dimension. J=round(1+rand), when rand is a random number in the range of [0, 1] and so $J\in \left\{1,2\right\}$. The idea of choosing any of the strategies is detected by $P_{s}$ and is done in the interval of [0, 1], which is again randomly generated. Depending on the second stage, the $j^{th}$ zebra’s updated position is indicated as $Y_{j}^{new,P_{2}}$. Before updating the $j^{th}$ zebra, the corresponding fitness value is identified as $F_{i}$. Based on the novel positions of the $j^{th}$ zebra, the respective fitness value is expressed by $F_{i}^{new,P_{2}}$. The updated position can replace the original position only if the new fitness value is better; otherwise, it remains the same.

### 3.3. GSA-PSO Algorithm

The GSA and PSO are hybridized together, the process of which is explained in the following section.

#### 3.3.1. GSA

Based on Newton’s gravity law and the laws of motion, GSA was developed [54]. With the aid of particle movements, the initial best solution is found by this algorithm in the entire group. Based on the gravity law, the progression of the mutual attraction within the particles is achieved. Depending on this rule, the particles can be effectively searched for in the process. With the movement of the particles, there is good improvement in the optimal solution and it can rise with the total number of iterations. We can assume there is a system which comprises Q particles in the entire search space, and it is represented as follows:

(38) $$H_{i}=\left(h_{i}^{1},\ldots ,h_{i}^{z},\ldots ,h_{i}^{n}\right)\;for\;i=1,2,\ldots ,Q$$

where the location of a particular particle is represented as $h_{i}^{z}$. Among the particles j and i, the gravitation at a particular iteration n is expressed as follows:

(39) $$F_{ij}^{z}(n)=H(n)\frac{M_{pasi}(n)\times M_{actj}(n)}{E_{ij}(n)+\varepsilon }\left(h_{j}^{z}(n)-h_{i}^{z}\left(n\right)\right)$$

where $M_{actj}(n)$ indicates the inertial masses of the active force particle j and $M_{pasi}(n)$ indicates the inertial masses of the passive force particle i. The Euclidean length is specified by $E_{ij}(n)$ and a small constant is specified by ε. At a particular iteration n, the attractional constant is expressed by H(n) and is mathematically modeled as

(40) $$H(n)=H_{0}e^{-\beta \frac{n}{T}}$$

The total amount of iterations is expressed by T. The β and $H_{0}$ are constant values and can have values ranging from [20 to 100], respectively. To enhance the stochastic properties of the algorithm, the total force $F_{i}^{z}(n)$ equals the sum of forces of all the other particles in the GSA and is represented as follows:

(41) $$F_{i}^{z}(n)=\sum_{j\neq i,j=1}^{Q}rand_{j}F_{ij}^{z}(n)$$

where the stochastic gradient is expressed as $rand_{j}$ and lies in the range of [0, 1]. For the particle i at n iterations, the acceleration is expressed by $j_{i}^{z}(n)$ and represented as follows:

(42) $$j_{i}^{z}(n)=\frac{F_{i}^{z}(n)}{M_{ii}(n)}$$

where, for the $i^{th}$ particle, the inertia mass is represented by $M_{ii}(n)$. The velocity and phase of the particle is renewed after every iteration and it is represented as follows:

(43) $$v_{i}^{z}(n+1)=rand_{i}\times v_{i}^{z}(n)+g_{i}^{z}(n)$$

(44) $$p_{i}^{z}(n)=p_{i}^{z}(n)+v_{i}^{z}(n+1)$$

Depending on the fitness value, the computation of inertia and gravity mass is as follows:

(45) $$m_{i}(n)=\frac{f_{i}(n)-\mathrm{worst}(n)}{best(n)-worst(n)}$$

(46) $$G_{i}(n)=\frac{m_{i}(n)}{\sum_{j=1}^{Q}m_{j}(n)}$$

where the fitness value of the $i^{th}$ particle is represented as $f_{i}(n)$ during the iteration process. For a maximization problem, the following equation is utilized in GSA as follows:

(47) $$best(n)=\underset{j\in 1,\ldots ,N}{\max}f_{j}(n)$$

#### 3.3.2. PSO

To obtain the optimal solution in the PSO, the movement of the birds flying is utilized thoroughly in the search space [55]. The optimal solution is nothing but the best particle and it is obtained along its path. In a target space, a population of Q particles is present with dimension z, where the $i^{th}$ particle indicates a vector with a specific dimension Z and is represented as follows:

(48) $$H_{i}=\left(h_{i1},h_{i2},\ldots ,h_{iZ}\right),i=1,2,\ldots ,Q$$

where the position of the $i^{th}$ particle is indicated by $h_{iZ}$. For the $i^{th}$ particle, the flight speed is indicated as follows:

(49) $$S_{i}=\left(s_{i1},s_{i2,\ldots ,}s_{iZ}\right),i=1,2,\ldots ,Q$$

where the $i^{th}$ particle speed is detailed by $s_{iZ}$. The $i^{th}$ particle searches for the best position and is denoted as $p_{best}$ and represented as follows:

(50) $$p_{best}=\left(p_{i1},p_{i2},\ldots ,p_{iZ}\right),i=1,2,\ldots ,Q$$

where the $i^{th}$ particle’s best position is represented as $p_{iZ}$. The best position for all the particles is represented as $g_{best}$ and expressed as follows:

(51) $$g_{best}=\left(p_{g1},p_{g2},\ldots ,p_{gZ}\right)$$

where the optimum value for all the particles is expressed as $p_{gZ}$. Once the optimal values are traced, then the speed and particles are renewed as follows:

(52) $$s_{iz}(n+1)=\theta \cdot s_{iz}(n)+b_{1}\cdot c_{1}\left(p_{iz}-h_{iz}(n)\right)+b_{2}\cdot c_{2}\left(p_{gz}-h_{iz}(n)\right)$$

(53) $$h_{iz}(n+1)=h_{iz}(n)+s_{iz}(n+1)$$

where $b_{1}$ and $b_{2}$ specify the study rate, and $c_{1}$ and $c_{2}$ specify the stochastic random process lying in the range of [0, 1].

#### 3.3.3. Hybridizing the GSA-PSO Algorithm

**Figure 3:**
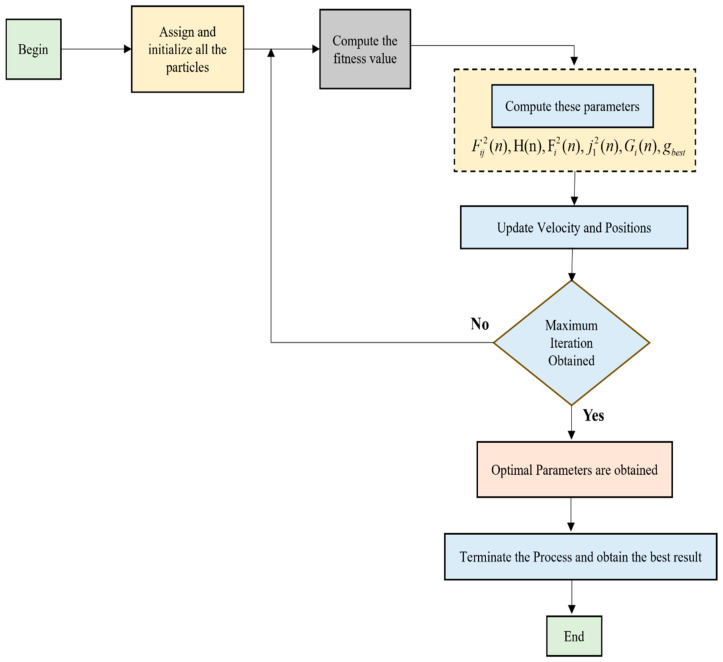
Simplified illustration of the GSA-PSO algorithm.

The inherent advantages of both PSO and GSA are combined; then, this hybrid algorithm is built. A simplified illustration of this is shown in Figure 3. The new updated speed and positions are modeled as follows:

(54) $$s_{iz}(n+1)=\theta \cdot s_{iz}(n)+b_{1}\cdot c_{1}\cdot k_{i}(n)+b_{2}\cdot c_{2}\left(p_{gz}-h_{iz}\left(n\right)\right)$$

(55) $$h_{iz}(n+1)=h_{iz}(n)+s_{iz}(n+1)$$

where the acceleration of $i^{th}$ particle is expressed as $k_{i}(t)$ during an iteration t. The overall procedure of GSA-PSO is as follows:

(1) All the particles are randomly initialized.
(2) The fitness evaluation function is constructed.
(3) The parameter value fed to every classifier is optimized by the feature selection optimization technique.
(4) The construction of fitness function is as follows:
  (56) $$fitness=\frac{1}{S}\sum_{i=1}^{S}\left(\frac{N-E}{N}\times 100\%\right)$$
  where the amount of data is represented by N and the entire error is denoted by E. S denotes the total number of subsets.
(5) A fitness value is attained for every iteration.
(6) Then, the fitness value of the present iteration is compared to the fitness value of the previous iteration and only the best fitness value is considered and updated.
(7) Compute and renew the values of $F_{ij}^{2}(n),H(n),F_{i}^{2}(n),j_{1}^{2}(n),G_{i}(n),g_{best}$ based on Equations (39)–(42), (46), and (51).
(8) Compute the speed of the particle based on (54).
(9) Compute the position of the particle based on (55).
(10) Implement step 2 to step 9 until the stopping criteria is met.
(11) Obtain the best solution and terminate the process.

## 4. Classifiers Utilized in This Work

In this study, we utilize four hybrid classifiers: the Collaborative Adaboost–ELM classification model, the CART-based Adaboost classification model, the hybrid weighted broad learning system-based Adaboost (HWBLSA) classifier, and a hybrid machine learning classification model with a soft voting technique.

### 4.1. Collaborative Adaboost–ELM Algorithm

This section explains the nuances of the ELM, Adaboost, and the Collaborative Adaboost–ELM classification model.

(A) ELM:

A widely used machine learning technique is ELM [56]. There is always a random distribution of the input weights and bias of the hidden layer in this algorithm and so it differs greatly from the conventional machine learning techniques. Also, there is no need to adjust these parameters often, which is one of its greatest advantages. A high learning speed is obtained by ELM and good performance is achieved often when using this technique. The basic details of ELM are as follows:

Assuming a dataset with N distinct samples $\left(p_{i},o_{i}\right)\in {ℜ}^{n}\times {ℜ}^{m}$, the output function can be represented for $\hat{N}$ hidden nodes and an activation function h(p), as follows:

(57) $$\sum_{i=1}^{\hat{N}}\beta_{i}h_{i}\left(p_{j}\right)=\sum_{i=1}^{\hat{N}}\beta_{i}h_{i}\left(w_{i}p_{j}+t_{i}\right)=q_{j},j=1,2,\ldots ,N$$

The threshold of the hidden layer is indicated as $t_{i}$ and the output is denoted as $q_{j}$. The output weights and input weights are specified as follows:

(58) $$\beta_{i}={\left[\beta_{i1},\beta_{i1},\ldots ,\beta_{im}\right]}^{N}$$

(59) $$w_{i}={\left[w_{i1},w_{i2},\ldots ,w_{in}\right]}^{N}$$

**Figure 4:**
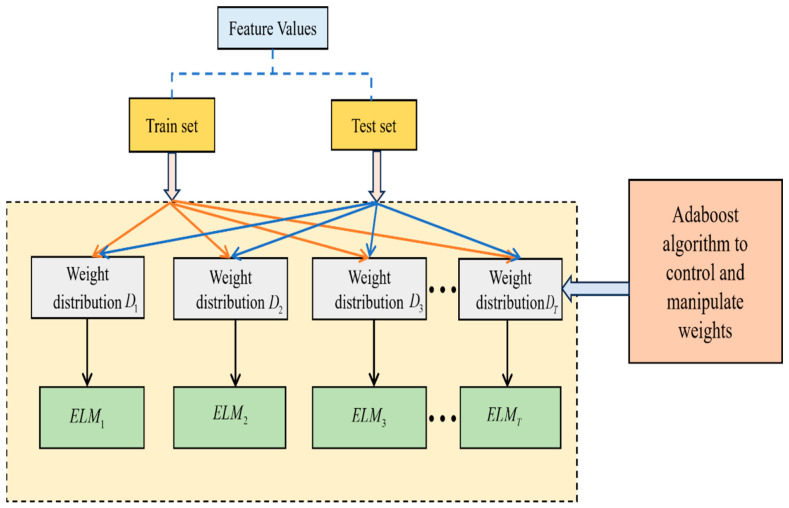
Simplified illustration of the Collaborative Adaboost–ELM model.

A zero error should be obtained between $q_{j}$ and $o_{j}$ parameters, and it is represented as follows:

(60) $$E={\sum_{j=1}^{N}\left(\sum_{i=1}^{\hat{N}}\beta_{i}h_{i}\left(w_{i}p_{j}+t_{i}\right)-o_{j}\right)}^{2}$$

(B) Adaboost:

The Adaboost algorithm is a successful pattern recognition algorithm, in which multiple weak predictions are hybrid so that a strong predictor can be established effectively [57]. For the samples, the distribution weights can be high when the error is maximum when training the Adaboost. Vice versa, the distribution weights can be low when the error is minimum when training the Adaboost. Depending on the distribution of new weight, the samples are trained so that the predicted output can be largely improved. The computation and procedure are as follows:

Step 1: Assume a sample set $W=\left\{\left.\left(p_{i},q_{i}\right)\right|i=1,2,\ldots ,N\right\}$. For the samples, the weight distribution is specified as $D_{t(i)},t=1,2,3,\ldots ,T$ at the $t^{th}$ iteration.
Step 2: At the initial iteration, the weight distribution is assigned as D1(i)=1N when t=1.
Step 3: Depending on the distribution of weights, the predicted output of $f_{t}\left(p_{i}\right)$ is computed.
Step 4: The forecasting error can be computed as
  (61) $$e_{t(i)}=\left|f_{t}\left(p_{i}\right)-q_{i}\right|,e_{t(i)}\in \left[0,1\right]$$
Step 5: The proportional error is computed as
  (62) $$\varepsilon_{t}=\sum_{i=1}^{N}D_{t(i)}e_{t(i)}$$
Step 6: The connection weights are computed as
  (63) wt=12log1βt
  where $\beta_{t}=\frac{\varepsilon_{t}}{\left(1-\varepsilon_{t}\right)}$.
Step 7: The weight distribution is now updated as
  (64) $$D_{t+1(i)}=\left(D_{t,(i)}\times \beta_{t}^{-\varepsilon_{t}}\right)/U_{t+1}$$
  (65) $$U_{t+1}=\sum_{i=1}^{N}D_{t+1,(i)}$$
Step 8: The final predictions after a certain number of T iterations is attained by
  (66) $$F\left(p\right)=\sum_{t=1}^{T}w_{t}f_{t}(p)$$

(C) Collaborative framework model of ELM–Adaboost:

The framework of this Collaborative Adaboost–ELM model is shown in Figure 4.

Step 1: The features are fed inside the collaborative framework model as test and training sets.
Step 2: To deal with the mathematical manipulation of the dataset, it is entirely normalized with the following equation:
  (67) $$Q_{t}^{\prime }=\frac{Q_{t}-Q_{\min}}{Q_{\max}-Q_{\min}}$$
  where $Q_{t}$ denotes the data present before normalization and $Q_{t}^{\prime }$ denotes the data obtained after normalization process. $Q_{\min}$ and $Q_{\max}$ indicate the minimum and maximum values of the original feature set.
Step 3: To establish multiple ELMs, the Adaboost algorithm is used, which helps to control the weight of various ELMs.
Step 4: The predicted output of ELMs is computed so that the relevant errors are calculated effectively.
Step 5: Once this is done, the weight distributions $D_{i}(i=1,2,\ldots ,T)$ are updated, respectively.
Step 6: The individual outputs of the ELMs are summarized successfully with the aid of connection weights.
Step 7: Finally, a good ensemble output is obtained in terms of the desired performance metric.

### 4.2. CART-Based Adaboost Classifier

To analyze Alzheimer’s disease classification, the CART-based Adaboost classifier is used. The performance of the classifier is improved by integrating CART with the Adaboost algorithm.

(A) CART procedure:

For regression and classification problems, one commonly used machine learning technique is the decision tree algorithm. A flowchart-like tree model of this type is initially generated so that the algorithm partition is generated successfully. A root node represents the start in the decision tree and then it is followed by child and leaf nodes so that it can have all the essential data required to train the model. The data are partitioned in a recursive manner and assessed by the tree structure so that the splitting criteria can be successful, as the goal is to obtain a leaf node. For the analysis of a decision tree, splitting criteria are highly necessary. CART is one such type of decision tree algorithm implemented to successfully prove the splitting criteria [58]. The Gini Index (GI) is utilized as the splitting criterion, which analyzes the impurity of a sample to a large extent. For a sample set S in a specific node, the GI is computed as follows:

(68) $$GI(S)=1-\sum_{c=1}^{C_{s}}p_{c}^{2}$$

**Figure 5:**
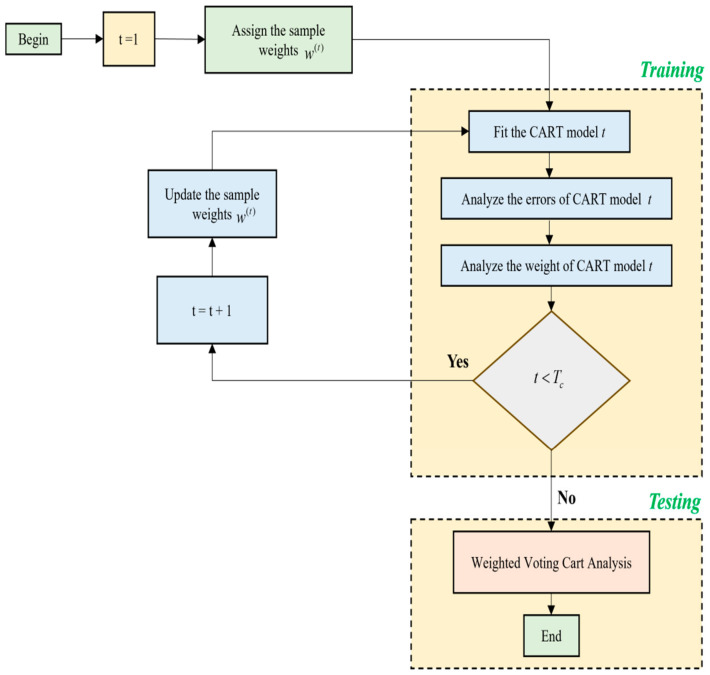
Simplified illustration of CART–Adaboost classifier.

To achieve high-purity samples, the GI should be small. In every child node, the weighted average of the GI is reduced by the splitting quality attribute. The maximization of ΔGI(S) is expressed as follows:

(69) $$\Delta GI(S,a_{t})=\mathrm{GI}(S)-\left[W_{L}.GI\left(S_{L}\right)+W_{R}.GI\left(S_{R}\right)\right]$$

where the number of categories is indicated as $C_{s}$ in the node and $a_{t}$ represents the attribute of the data split mode. The proportion of the $c^{th}$ category in the node is represented by $p_{c}$. The difference in impurity before and after a split is indicated by $\Delta GI(S,a_{t})$. The sample set of the left child node is represented by $S_{L}$ and the proportion of the left child node is represented by $W_{L}$. The sample set of the right child node is represented by $S_{R}$, and the proportion of the right child node is represented by $W_{R}$. In the CART technique, there is no necessity to choose independent variables initially, which represents one of the greatest advantages of using the CART technique. When every node undergoes splitting, the best variables are chosen and utilized. When the correlation between the input and output parameters is not known, CART can be utilized as it interprets the results quantitatively and qualitatively and so it is used for both regression and classification purposes.

(B) Implementation of CART–Adaboost classifier:

A single CART model cannot perform exceedingly well as it has some constraints due to the limited structure of the generated trees. Good accuracy is not obtained if the tree structure is very simple and so ensemble techniques are utilized here in the form of the CART-based Adaboost classification model, where the base learner of the Adaboost algorithm is found by the CART procedure. A simplified illustration of this procedure is shown in Figure 5. To improve the accuracy of the model, the sample weights are adjusted continuously during training of the CART–Adaboost classification process. To obtain the ultimate prediction result, the weightage of every output in CART is given a lot of importance in this classification model.

### 4.3. Hybrid Weighted Broad Learning System-Based Adaboost (HWBLSA) Classifier

The concept of the weighted broad learning system is integrated with the Adaboost classifier and it forms an HWBLSA classifier.

(A) Concept of Weighted Broad Learning System

There are three layers present in the broad learning system (BLS), and the enhancement nodes and feature nodes are contained in the hidden layer [59]. The training samples are represented as $\left\{\left.p_{i}\right|p_{i}\in R^{M}\right\}$ and its respective label is expressed as $\left\{\left.q_{i}\right|q_{i}\in R^{C}\right\}$. From the feature nodes, the input samples P are proceeded as follows:

(70) $$J_{i}=\varphi_{i}\left(PW_{ei}+\beta_{ei}\right),i=1,2,\ldots ,n$$

where the mapping function is indicated as $\varphi_{i}$. The bias is specified as $\beta_{ei}$ and the generated weight is represented in a random manner as $W_{ei}$. The total number of ‘n’ mapping windows is concatenated in the row direction and now, the feature nodes can be specified as follows:

(71) $$J^{n}=\left[J_{1},J_{2},\ldots ,.J_{n}\right]$$

Through efficiently managing feature nodes, enhanced nodes can be attained as follows:

(72) $$E_{k}=\xi_{k}\left(J^{n}W_{ek}+\beta_{ek}\right),k=1,2,3,\ldots ,m$$

where the activation function is represented as $\xi_{k}\left(\cdot \right)$. The randomly generated bias is specified as $\beta_{ek}$ and the randomly generated weight is specified as $W_{ek}$. The attainment of enhancement nodes can be expressed as follows:

(73) $$E^{m}=\left[E_{1},E_{2},\ldots ,E_{m}\right]$$

The following relationship has been developed by BLS and it is expressed as follows:

(74) $$\left\{\begin{array}{c} Q=UW\\ U=\left[\left.J^{n}\right|E^{m}\right] \end{array}\right.$$

where the true value of samples is represented as U. The weight W expresses the output weight and is directly connected to the output layer. The weight W serves as a bridge between the enhancement and feature nodes and can be solved by ridge regression, as follows:

(75) $$\underset{W}{\arg\min}:{\left\|UW-Q\right\|}_{2}^{\sigma_{1}}+\lambda {\left\|W\right\|}_{2}^{\sigma_{2}}$$

where the regularization parameter is represented as λ.

For the datasets whose samples are imbalanced, an accurate prediction is not possible using general models. So, cost-sensitive techniques are used to improvise them and, with the aid of cost matrices, the classification issue can be solved. The original cost-sensitive matrix is expressed as follows:

(76) $$M=\left[\begin{array}{cccc} 0 & M_{12} & \ldots & M_{1L}\\ M_{21} & 0 & \ldots & M_{2L}\\ \vdots & \vdots & \vdots & \vdots \\ M_{L1} & M_{L2} & \ldots & 0 \end{array}\right]$$

where $M_{ij}$ expresses the risk of misclassification of a sample $p_{i}$ and $p_{j}$. When the cost of the matrix increases, the error discrimination can be largely reduced. For the BLS, the optimization of the objective function is expressed as follows:

(77) $$\underset{W}{\arg\min}:{\left\|UW-Q\right\|}_{2}^{2}+\lambda {\left\|W\right\|}_{2}^{2}$$

BLS incorporates the cost-sensitive matrix and so it is expressed as follows:

(78) $$\underset{W}{\arg\min}:M{\left\|UW-Q\right\|}_{2}^{2}+\lambda {\left\|W\right\|}_{2}^{2}$$

where the cost-sensitive matrix M can be generated in a random manner. The classification error can be alleviated easily when using this method and so imbalanced datasets can be dealt with very well. The training error is accumulated iteratively and the majority of the class samples will be trained easily. The samples with the majority class are generally favored by the classification results. The samples which have more error space are generally considered as minority class samples. A huge weight is picked for the minority class samples and a small weight is picked for the majority class samples. Over time, a training bias can be managed well between the minority and majority samples. A weight is multiplied to every sample so that a good training bias is achieved and is expressed as follows:

(79) $$Z_{ii}=\left\{\begin{array}{l} \frac{\varepsilon }{class(p_{i})},class\left(p_{i}\right)>average(class)\\ \frac{1}{class\left(p_{i}\right)},\;otherwise \end{array}\right.$$

where 0<ε≤1 is the original norm condition. The total number of samples is expressed by $class\left(p_{i}\right)$. The average of the entire classes is expressed by the average(class) parameter. The extent of continuous equilibrium of the sample is measured by the term $Z_{ii}$. To the samples of the majority class, the boundary is extended and its value is always small in between the minority and majority classes’ samples, so that good accuracy can be achieved. The objective function can be modified by the addition of weights $Z_{ii}$ so that it is expressed as follows:

(80) $$\underset{W}{\arg\min}:V{\left\|UW-Q\right\|}_{2}^{2}+\lambda {\left\|W\right\|}_{2}^{2}$$

where V=ZM, $Z=diag\left(Z_{ii}\right)$, k=1,2,3,…,m.

The ultimate output weight of the WBLS is expressed as follows:

(81) $$W={\left(\lambda I+U^{T}VU\right)}^{-1}U^{T}VQ$$

(B) HWBLSA classifier

Multiple individual classifiers are combined so that a robust classifier can be created; in machine learning terms, this is known as ensemble learning. For the various classifiers, the diversity can be leveraged so that the classification performance can be greatly improved when compared to that of a single classifier. Bagging, boosting, and AdaBoost are some commonly used ensemble algorithms. The weak classifiers are trained iteratively by this AdaBoost algorithm so that their predictions are hybrid, allowing them to obtain a strong classifier. Weight can be adjusted adaptively by AdaBoost so that higher weights can be assigned to misclassified samples. To the samples which are currently classified, lower weights can be assigned in the process. During every iteration, the samples are challenged so that the overall accuracy is enhanced. Through a process called weighted voting, the weak classifiers are combined by Adaboost so that their collective predictions are used and a versatile classifier is created. The weighted sum or contributions of the independent classifiers are considered so that the final decision can be made and the accurate ones can be given more priority. An exponential loss function is utilized on the sample set, which is expressed as follows:

(82) $$\alpha_{m}=\frac{1}{2}\ln\frac{1-e_{m}}{e_{m}}+\ln\left(C_{at}-1\right)$$

where $C_{at}$ denotes the number of classification categories. To assess the base classifiers, the sample set type is analyzed and the probability magnitude is measured directly in this process. In the dataset, the imbalance characteristics are tried and balanced as much as possible so that the minority classes can be better discriminated. The distribution characteristics are analyzed well by suitable weights so that a higher accuracy is obtained. This is achieved by hybridizing the Adaboost concept with the WBLS. The base classifier considered is WBLS, and after several iterations using multiple base classifiers, it can ultimately be integrated as a robust HWBLSA classifier.

(C) HWBLSA implementation

The weights wi=1n are initialized by the HWBLSA classifier. The base classifier is iterated successfully a total of m times, where the inputs are given as $P=\left(p_{1},\ldots ,p_{n}\right)$ and $Q=\left(q_{1},\ldots ,q_{n}\right)$, respectively, from m=1,2,…,M. WBLS is considered the initial base classifier and an approximate probabilistic model is generated as follows:

(83) $$P_{l}^{\left(m\right)}\left(p_{i}\right)=P_{w}\left(S\left(q_{i}\right)=\left.l\right|p\right),l=1,2,\ldots ,L$$

With the help of a probabilistic model, the new classifier can be synthesized as follows:

(84) $$h_{l^{\prime }}^{\left(m\right)}\left(p_{i}\right)=\left(L-1\right)\left[\log P_{l^{\prime }}^{\left(m\right)}(p)-\frac{1}{L}\sum_{l=1}^{L}\log P_{l}^{\left(m\right)}(p)\right]$$

The weights are updated as follows:

(85) $$w_{i}=w_{i}\cdot \exp\left(-\frac{L-1}{L}q_{i}^{T}\left[\log P_{1}^{\left(m\right)},\ldots ,\log P_{l}^{\left(m\right)}\right]\right)$$

where the weights $w_{i}$ are normalized utilizing $w_{i}=\left.w_{i}\right|\sum_{i=1}^{n}w_{i}$.

Until the required number of iterations are obtained, the above process is continued. For the classifier, the final output $C_{out}$ is represented as follows:

(86) $$C_{out}(p)=avg\underset{l}{\max}\sum_{m=1}^{M}h_{l}^{\left(m\right)}(p)$$

### 4.4. Hybrid Machine Learning Models with Soft Voting Technique

Machine learning plays a major role in the prompt detection and analysis of Alzheimer’s disease using EEG signals. Correlations and patterns can be discerned easily by machine learning models and so it is highly useful in analyzing any disease. The collective strength of the different machine learning models can be leveraged; thus, a good classification accuracy can be achieved with these ensemble learning techniques. These hybrid models can be fine-tuned by adjusting the hyperparameters depending on the necessity of the datasets and feature selection algorithms used. In this work, Logistic Regression (LR), SVM, Random Forest (RF), and KNN are used and hybridized together with a soft voting technique.

**Figure 6:**
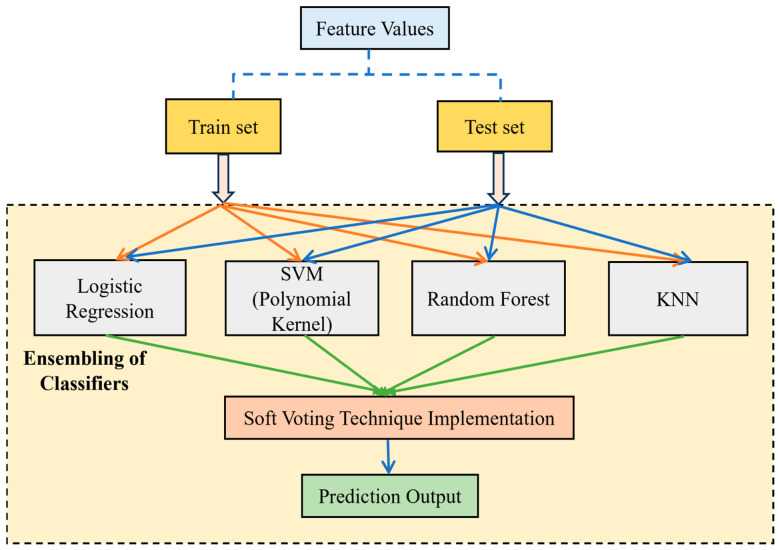
Simplified illustration of the hybrid machine learning model with soft voting technique.

By means of employing the soft voting criteria, the final prediction can be assessed depending on the class with the largest probability [60]. When these models are hybridized together, the bias and variance can be reduced and so the overall robustness and accuracy can be improved well. A reliable and balanced decision can be made when employing the soft voting technique, as it ensures that there are very few or negligible misclassifications since multiple classifiers are engaged in determining the output. Therefore, an accurate and stable prediction analysis can be performed; thus, it is employed widely in the medical diagnosis of many disorders, outperforming the standalone classifiers or conventional models. Depending on the inherent characteristics of machine learning models, some can struggle with overfitting problems, an inability to deal with complex patterns, and interpretability issues, but once all the classifiers are hybrid and a soft voting strategy is implemented, the strength of these classifiers is then leveraged and a solid output can be bought out, implying a very good performance. Figure 6 shows a simplified illustration of the proposed hybrid machine learning model with the soft voting technique.

## 5. Results and Discussion

To analyze the proposed framework, a publicly available EEG signal dataset for detecting and classifying AD was utilized [61]. EEG signals were acquired from nine participants (eight healthy individuals and one with AD). The participants were made to sit comfortably in front of a monitor in a dimly lit room. A good distance was maintained between the participants when analyzing the experiments. A cathode ray tube (CRT) monitor was used to present a visual stimulus with the help of E-prime 2 software so that eye movements could be monitored easily. Each trial started with a fixation cross that was followed by a stimulus presentation accompanied by a warning tone. The participants completed a discrimination task, and the stimulus was presented for 300 ms. In experiment 1, the participants were asked to indicate whether the presented stimulus was a house, a face, or a scrambled image. In experiment 2, the stimuli comprised visual faces with fearful or neutral expressions, and in experiment 3, the stimuli were unfamiliar or very famous faces. Detecting amnesia or agnosia in the broader context of face recognition deficits in patients with AD using EEG signals was the main intention of the work, and so for convenience, experiment 1 employed 1225 healthy classes and 325 AD sample values and experiment 2 utilized 1200 healthy classes and 350 AD sample values. The overall dataset was quite small and imbalanced, so the proposed work was versatile, with no need to incorporate deep learning as the overall data size was very small. A repeated 10-fold cross-validation technique was employed throughout the work for the analysis. For performing the experiment, MATLAB R2022a software installed on a system with 32 GB of main memory, an i5 processor, and the Windows 11 Operating System was utilized.

As far as the KPLS technique is concerned, a Gaussian Kernel was utilized, and for the Kriging Model, the length scale was set to 0.5 and the overall variance was set to 0.2 for the entire process. The number of neighbors used in the Isomap algorithm was set as 10 and for the K-means clustering technique, the number of clusters was set to around 20 in the experiment. For the CSO-RSO algorithm, the parameters of CSO were set as follows: the population size was 100, the step size was 0.5, the Levy exponent was 1.5, and the probability of discovery was assigned in the range of [0, 1]. For RSO, the population size was again set as 100 and the total number of iterations was expressed as 200 and the number of dimensions was assigned depending on the distance and search space. The hunting mode of rats was set in the range of [0, 1]. For ZOA, the population size was set as 50, the maximum iteration was set as 100, and the random number range was set in the order of [0, 1], which helped model the movement of the zebras. For the hybrid GSA-PSO algorithm, the parameters of GSA were as follows: the population size was set as 100 and the gravitational constant was set as 0.5, the maximum iterations were set to 200, and the acceleration coefficients were set in the range of [0, 10] depending on the search space boundaries. For PSO, the population size was assigned as 100 and the inertia weight was initially set to 0.5. The cognitive and social coefficient values were assigned as 0.2 and 0.4, respectively, and the maximum number of iterations was set to 200 in the experiment. For the LR classifier, the multinomial class was selected and for SVM, the Kernel used in this work was polynomial. The value of C utilized in the SVM classifier was set to 2 so that fewer errors were obtained in the process and a good regularization could be achieved. For RF, the number of estimators used is 250 and the max_depth parameter set was 100, and for the KNN classifier, the total number of weights set was 5. The maximum iteration limit of 500 was set in the conventional machine learning classification process so that a good convergence was achieved.

According to the analyses completed on the data for experiment 1, a high classification accuracy of 92.22% is obtained when Kriging Model feature extraction is combined with CSO feature selection and classified with the CART–Adaboost classifier (Table 1); a high classification accuracy of 88.23% is obtained when KPLS Model feature extraction is combined with RSO feature selection and classified with the HWBLSA classifier (Table 2); a high classification accuracy of 97.89% is obtained when Kriging Model feature extraction is combined with hybrid CSO-RSO feature selection and classified with the ELM–Adaboost classifier (Table 3); a high classification accuracy of 95.98% is obtained when KPLS Model feature extraction is combined with ZOA feature selection and classified with the HWBLSA classifier (Table 4); a high classification accuracy of 93.89% is obtained when Kriging Model feature extraction is combined with GSA feature selection and classified with the ELM–Adaboost classifier (Table 5); a high classification accuracy of 92.56% is obtained when Kriging Model feature extraction is combined with PSO feature selection and classified with the HWBLSA classifier (Table 6); and lastly, a high classification accuracy of 96.56% is obtained when Kriging Model feature extraction is combined with hybrid GSA-PSO feature selection and classified with the HWBLSA classifier (Table 7).

According to the analysis of the data for experiment 2, a high classification accuracy of 94.89% is obtained when KPLS feature extraction is combined with CSO feature selection and classified with the HWBLSA classifier (Table 8). A high classification accuracy of 91.09% is obtained when RSO feature extraction is combined with RSO feature selection and classified with the HWBLSA classifier (Table 9). A high classification accuracy of 98.65% is obtained when KPLS feature extraction is combined with hybrid CSO-RSO feature selection and classified with the ELM–Adaboost classifier (Table 10). A high classification accuracy of 96.44% is obtained when KPLS Model feature extraction is combined with ZOA feature selection and classified with the ELM–Adaboost classifier (Table 11). Table 12 shows that a high classification accuracy of 91.78% is obtained when KPLS Model feature extraction is combined with GSA feature selection and classified with a hybrid classifier with the soft computing technique (Table 12). A high classification accuracy of 90.78% is obtained when Kriging Model feature extraction is combined with PSO feature selection and classified with the ELM–Adaboost classifier (Table 13). Lastly, a high classification accuracy of 98.71% is obtained when Kriging Model feature extraction is combined with hybrid GSA-PSO feature selection and classified with the ELM–Adaboost classifier (Table 14).

**Figure 7:**
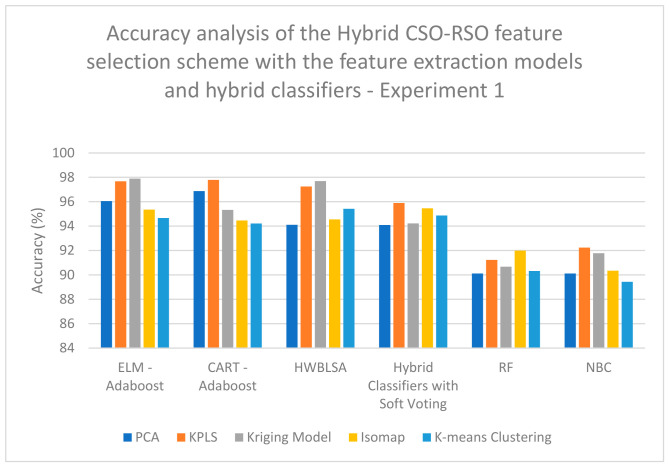
Performance analysis of accuracy of the hybrid CSO-RSO feature selection scheme with the feature extraction models and hybrid classifiers—Experiment 1.

**Figure 8:**
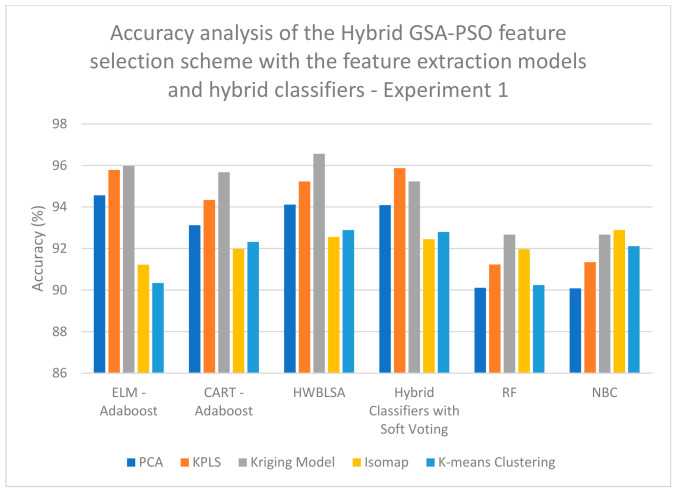
Performance analysis of accuracy of the hybrid GSA-PSO feature selection scheme with feature extraction models and hybrid classifiers—experiment 1.

**Figure 9:**
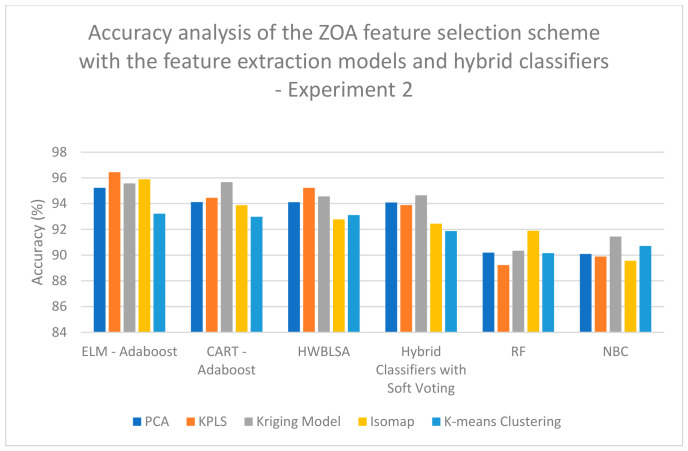
Performance analysis of accuracy of the ZOA feature selection scheme with the feature extraction models and hybrid classifiers—experiment 2.

**Figure 10:**
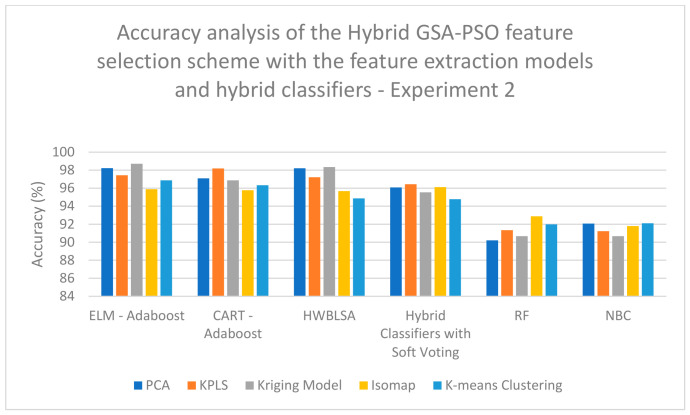
Performance analysis of accuracy of the hybrid GSA-PSO feature selection scheme with the feature extraction models and hybrid classifiers—experiment 2.

On examining Figure 7, it is evident that a high classification accuracy of 97.89% is obtained when Kriging Model feature extraction is combined with hybrid CSO-RSO feature selection and classified with the ELM–Adaboost classifier when analyzing the data for experiment 1. On examining Figure 8, a high classification accuracy of 96.56% is obtained when Kriging Model feature extraction is combined with hybrid GSA-PSO feature selection and classified with the HWBLSA classifier when analyzing the data for experiment 1. In Figure 9, it can be seen that a high classification accuracy of 96.44% is obtained when KPLS Model feature extraction is combined with ZOA feature selection and classified with ELM–Adaboost classifier when analyzing the data for experiment 2. When Figure 10 is examined, a high classification accuracy of 98.67% is obtained when Kriging Model feature extraction is combined with hybrid GSA-PSO feature selection and classified with the ELM–Adaboost classifier when the analysis is completed on the data from experiment 2.

As far as statistical analysis is concerned, when a two-sided Wilcoxon test was executed, a good confidence level was achieved for all the features. When the Friedman test was executed, the feature values developed a good variation amongst themselves, thereby proving fit for classification. Since only machine learning techniques were used in the experiment, the overall computational complexity was attained at $O\left(n^{3}\log n\right)$ only. The computational time for the proposed models was calculated as follows:

As is evident from Table 15, a low computational time of 5.003 s was obtained for the combination of KPLS + hybrid CSO-RSO feature selection + ELM–Adaboost classification model. The next best computational time of 5.609 s was obtained for the combination of the Kriging Model + hybrid GSA-PSO feature selection + ELM–Adaboost classification model. A comparatively high computational time of 10.914 s has been obtained for the combination of KPLS + RSO feature selection + HWBLSA classification model.

### 5.1. Comparison with Previous Works

Only one study has been reported that has used this specific dataset previously for AD detection and classification for the sake of performance comparison. However, a few important results for AD detection with other datasets are discussed for the readers’ detailed understanding. In reference [15], 20 healthy controls and 34 patients with AD were used and spectral feature extraction with the SVM technique was employed; the authors reported a classification accuracy of 88.1%. In [17], 120 healthy controls and 175 patients with AD were used and electromagnetic tomography with SVM was employed; a classification accuracy of 95% was reported. In [21], 24 healthy and 24 patients with AD were analyzed and the method employed was Pearson’s correlation with CNN; 100% accuracy was achieved. In [25], 11 healthy controls and 12 patients with AD were used and the method employed was a novel primate brain pattern with KNN classifier; the authors obtained a classification accuracy of 100%. In [35], eight healthy controls and one patient with AD were analyzed and the method employed the Lattice 123 concept with standard machine learning classifiers; the analysis showed a classification accuracy of 98.37% for experiment 1 and of 99.62 for experiment 2. We compared our results to those obtained by the authors of [35], as we used a similar dataset to that used by them and the results were computed and compared only for experiments 1 and 2. The proposed framework showed that the best results were as follows: The highest classification accuracy of 98.71% was obtained when Kriging Model feature extraction was combined with hybrid GSA-PSO feature selection and classified with the ELM–Adaboost classifier. This is according to the analysis of the dataset for experiment 2. The second highest classification accuracy of 98.65% is obtained when KPLS feature extraction is combined with hybrid CSO-RSO feature selection and classified with ELM–Adaboost classifier when the analysis is done for experiment 2 of the dataset. The third highest classification accuracy, of 97.89%, was obtained when Kriging Model feature extraction was combined with hybrid CSO-RSO feature selection and classified with ELM–Adaboost classifier when the data for experiment 1 were analyzed. The fourth highest classification accuracy of 96.56% was obtained when Kriging Model feature extraction was combined with hybrid GSA-PSO feature selection and classified with the HWBLSA classifier when the analysis is completed for experiment 1 of the dataset. The obtained results show good quality in terms of classification accuracy and seem to be robust and versatile, and so this framework has the potential to be applied to analyze other neurological disorders as well.

### 5.2. Study Limitations 

For an ensemble model involving feature extraction, feature selection using standard methods, and biomimetics-based models followed by classification using conventional and hybrid machine learning models, one must pay careful attention to every module to avoid producing erroneous results. When dealing with feature extraction, issues may arise due to generalization, lack of interpretability, information loss, and computational intensity; thus, careful attention must be paid to it. Also, when dealing with feature selection, problems may occur such as a high cardinality bias, an inability to handle multicollinearity issues, overfitting issues, subjective bias, and unclear causality; therefore, appropriate measures must be taken when selecting efficient features. Any time the concept of biomimetics is used in research, various factors must be analyzed, such as sustainability issues, evolution constraints, knowledge gaps, complexity measures, and application bottlenecks. Thus, the application of biomimetics in certain fields needs careful analysis and experimentation. Finally, when machine learning is used, factors such as algorithm bias, computational cost and complexity, and time, as well as its ability to adapt dynamically, must be considered carefully when analyzing the experiment. A high level of analytical logic is always required when handling ensemble models, as hybridization and combinations are employed to ensure that the best results are consistently produced.

## 6. Conclusions and Future Work

AD is a neurological disease and patients suffering from it exhibit many symptoms, including memory loss, hallucinations, and the inability to perform basic daily tasks. Various genetic and environmental factors, as well as trauma and age, among others, contribute to the development of this disease. There is no specific or definite diagnostic test for AD; based on certain biomarkers and the clinician’s assessment, a treatment is decided upon. Artificial intelligence (AI)-based automated detection of AD assisted by EEG signals is widely used in the scientific research community. In this work, a consolidated framework is proposed that employs very interesting feature selection techniques and hybrid classifiers along with feature extraction schemes, and it is tested on an AD database. Following analysis, the proposed framework showed that the highest classification accuracy, at 98.71%, is achieved when Kriging Model feature extraction is combined with hybrid GSA-PSO feature selection and classified with the ELM–Adaboost classifier. The second highest classification accuracy, of 98.65%, is obtained when KPLS feature extraction is combined with hybrid CSO-RSO feature selection and classified with the ELM–Adaboost classifier. Future research should aim to modify the feature selection schemes and incorporate hybridization in the classification methods, so that a much higher accuracy can be achieved. Future studies should also aim to improve the overall model in terms of stability, reliability, and overall versatility, such that it can be applied to a variety of other biosignal processing and medical image processing datasets to analyze other neurological disorders and health issues.

## Figures and Tables

**Table 1:** Analysis of accuracy of the CSO feature selection scheme with the feature extraction models and hybrid classifiers—experiment 1.

	ELM–Adaboost	CART–Adaboost	HWBLSA	Hybrid Classifiers with Soft Voting	RF	NBC
**PCA**	90.46	87.56	91.02	89.02	84.23	83.23
**KPLS**	91.22	89.23	92.09	91.33	85.32	84.03
**Kriging Model**	91.22	92.22	91.03	90.11	85.56	84.56
**Isomap**	89.34	89.19	91.23	90.21	83.46	83.44
**K-means Clustering**	89.01	87.34	90.23	91.22	81.48	84.36

**Table 2:** Analysis of accuracy of the RSO feature selection scheme with the feature extraction models and hybrid classifiers—experiment 1.

	ELM–Adaboost	CART–Adaboost	HWBLSA	Hybrid Classifiers with Soft Voting	RF	NBC
**PCA**	88.04	85.09	86.11	88.11	82.02	80.11
**KPLS**	87.56	85.98	88.23	87.23	81.44	82.26
**Kriging Model**	86.77	84.34	88.07	88.01	82.79	81.98
**Isomap**	87.89	85.56	87.86	87.72	81.97	80.88
**K-means Clustering**	87.12	84.87	86.34	86.86	80.22	82.21

**Table 3:** Analysis of accuracy of the hybrid CSO-RSO feature selection scheme with the feature extraction models and hybrid classifiers—experiment 1.

	ELM–96.	CART–Adaboost	HWBLSA	Hybrid Classifiers with Soft Voting	RF	NBC
**PCA**	96.05	96.87	94.11	94.09	90.11	90.11
**KPLS**	97.67	97.79	97.25	95.89	91.23	92.24
**Kriging Model**	97.89	95.33	97.69	94.22	90.67	91.78
**Isomap**	95.35	94.46	94.55	95.46	91.99	90.34
**K-means Clustering**	94.66	94.21	95.42	94.87	90.32	89.43

**Table 4:** Analysis of accuracy of the ZOA feature selection scheme with the feature extraction models and hybrid classifiers—experiment 1.

	ELM–Adaboost	CART–Adaboost	HWBLSA	Hybrid Classifiers with Soft Voting	RF	NBC
**PCA**	94.23	93.12	94.78	93.89	90.09	91.21
**KPLS**	94.45	94.44	95.98	92.90	90.88	89.33
**Kriging Model**	93.77	93.67	94.11	94.54	89.13	88.46
**Isomap**	92.87	92.90	93.25	93.22	88.65	89.78
**K-means Clustering**	92.34	91.22	92.78	91.15	87.32	90.42

**Table 5:** Analysis of accuracy of the GSA feature selection scheme with the feature extraction models and hybrid classifiers—experiment 1.

	ELM–Adaboost	CART–Adaboost	HWBLSA	Hybrid Classifiers with Soft Voting	RF	NBC
**PCA**	92.56	91.89	92.09	92.12	89.21	88.34
**KPLS**	92.77	91.09	92.98	90.35	89.33	87.78
**Kriging Model**	93.89	92.33	91.44	92.78	88.57	87.66
**Isomap**	90.97	89.21	92.46	92.98	87.86	88.54
**K-means Clustering**	91.11	90.46	90.78	90.21	88.11	89.12

**Table 6:** Analysis of accuracy of the PSO feature selection scheme with the feature extraction models and hybrid classifiers—experiment 1.

	ELM–Adaboost	CART–Adaboost	HWBLSA	Hybrid Classifiers with Soft Voting	RF	NBC
**PCA**	91.21	90.09	92.11	91.12	87.09	87.11
**KPLS**	90.34	90.23	91.23	89.23	86.99	86.23
**Kriging Model**	90.67	90.56	92.56	90.44	88.82	85.67
**Isomap**	89.88	88.89	88.89	89.58	89.34	87.97
**K-means Clustering**	89.98	88.12	87.78	91.99	85.65	88.22

**Table 7:** Analysis of accuracy of the Hybrid GSA-PSO feature selection scheme with the feature extraction models and hybrid classifiers—experiment 1.

	ELM–Adaboost	CART–Adaboost	HWBLSA	Hybrid Classifiers with Soft Voting	RF	NBC
**PCA**	94.56	93.12	94.11	94.09	90.11	90.08
**KPLS**	95.78	94.34	95.23	95.87	91.23	91.34
**Kriging Model**	95.98	95.67	96.56	95.23	92.67	92.67
**Isomap**	91.22	91.98	92.56	92.45	91.96	92.89
**K-means Clustering**	90.34	92.32	92.89	92.80	90.24	92.11

**Table 8:** Analysis of accuracy of the CSO feature selection scheme with feature extraction models and hybrid classifiers—experiment 2.

	ELM–Adaboost	CART–Adaboost	HWBLSA	Hybrid Classifiers with Soft Voting	RF	NBC
**PCA**	91.23	92.34	93.09	91.09	85.09	85.21
**KPLS**	92.45	91.56	94.89	92.98	86.89	85.34
**Kriging Model**	93.67	92.89	92.96	91.24	86.77	86.67
**Isomap**	91.87	90.87	92.78	91.56	85.54	87.89
**K-means Clustering**	91.11	89.11	92.45	92.87	83.12	88.43

**Table 9:** Analysis of accuracy of the RSO feature selection scheme with feature extraction models and hybrid classifiers—experiment 2.

	ELM–Adaboost	CART–Adaboost	HWBLSA	Hybrid Classifiers with Soft Voting	RF	NBC
**PCA**	89.21	88.09	89.98	91.09	84.12	83.11
**KPLS**	88.33	88.46	89.78	90.89	83.45	84.25
**Kriging Model**	88.46	87.87	90.33	87.66	83.78	82.78
**Isomap**	89.78	88.32	88.45	88.34	84.66	83.93
**K-means Clustering**	88.98	88.68	87.78	89.78	82.43	84.12

**Table 10:** Analysis of accuracy of the hybrid CSO-RSO feature selection scheme with the feature extraction models and hybrid classifiers—experiment 2.

	ELM–Adaboost	CART–Adaboost	HWBLSA	Hybrid Classifiers with Soft Voting	RF	NBC
**PCA**	97.45	95.21	95.09	95.11	91.21	91.08
**KPLS**	98.65	96.34	96.98	94.23	90.22	92.34
**Kriging Model**	97.89	96.55	97.78	94.56	91.42	90.67
**Isomap**	96.84	95.68	95.99	96.87	92.74	91.11
**K-means Clustering**	95.55	94.98	94.32	94.55	91.09	91.57

**Table 11:** Analysis of accuracy of the ZOA feature selection scheme with the feature extraction models and hybrid classifiers—experiment 2.

	ELM–Adaboost	CART–Adaboost	HWBLSA	Hybrid Classifiers with Soft Voting	RF	NBC
**PCA**	95.23	94.12	94.11	94.09	90.19	90.09
**KPLS**	96.44	94.45	95.23	93.89	89.23	89.89
**Kriging Model**	95.57	95.67	94.56	94.65	90.34	91.44
**Isomap**	95.89	93.88	92.78	92.44	91.89	89.56
**K-means Clustering**	93.22	92.98	93.11	91.87	90.15	90.71

**Table 12:** Analysis of accuracy of the GSA feature selection scheme with the feature extraction models and hybrid classifiers—experiment 2.

	ELM–Adaboost	CART–Adaboost	HWBLSA	Hybrid Classifiers with Soft Voting	RF	NBC
**PCA**	90.34	89.78	91.11	90.09	87.21	87.12
**KPLS**	90.56	88.98	90.24	91.78	86.44	86.46
**Kriging Model**	89.87	91.22	90.77	90.33	87.67	88.87
**Isomap**	88.11	88.35	89.89	90.46	85.98	86.44
**K-means Clustering**	89.26	90.97	91.63	88.87	86.23	87.22

**Table 13:** Analysis of the accuracy of PSO feature selection scheme with the feature extraction models and hybrid classifiers—experiment 2.

	ELM–Adaboost	CART–Adaboost	HWBLSA	Hybrid Classifiers with Soft Voting	RF	NBC
**PCA**	88.34	87.09	90.21	90.09	86.09	86.01
**KPLS**	89.66	88.77	90.33	90.62	85.78	85.23
**Kriging Model**	90.78	89.46	89.65	89.56	86.55	87.67
**Isomap**	87.62	88.25	88.73	89.11	85.13	87.99
**K-means Clustering**	87.24	89.66	90.13	88.68	85.22	86.86

**Table 14:** Analysis of accuracy of the hybrid GSA-PSO feature selection scheme with the feature extraction models and hybrid classifiers—experiment 2.

	ELM–Adaboost	CART–Adaboost	HWBLSA	Hybrid Classifiers with Soft Voting	RF	NBC
**PCA**	98.23	97.09	98.21	96.09	90.21	92.07
**KPLS**	97.44	98.19	97.22	96.44	91.34	91.23
**Kriging Model**	98.71	96.87	98.35	95.54	90.67	90.68
**Isomap**	95.89	95.78	95.67	96.12	92.88	91.79
**K-means Clustering**	96.87	96.33	94.87	94.78	91.98	92.11

**Table 15:** Computational time analysis for the best-performing models.

Year	Authors	Methods	Computational Time
2026	Proposed works	Experiment 1: Kriging Model + CSO feature selection + CART–Adaboost model	8.012 s
		Experiment 1: KPLS + RSO feature selection + HWBLSA model	10.914 s
		Experiment 1: Kriging Model + Hybrid CSO-RSO feature selection + ELM–Adaboost model	5.781 s
		Experiment 1: KPLS + ZOA feature selection + HWBLSA model	6.887 s
		Experiment 1: Kriging Model + GSA feature selection + ELM–Adaboost model	7.812 s
		Experiment 1: Kriging Model + PSO feature selection + HWBLSA model	8.004 s
		Experiment 1: Kriging Model + hybrid GSA-PSO feature selection + HWBLSA model	6.108 s
		Experiment 2: KPLS + CSO feature selection + HWBLSA model	7.983 s
		Experiment 2: PCA + RSO feature selection + hybrid classifiers with soft voting model	9.136 s
		Experiment 2: KPLS + hybrid CSO-RSO feature selection + ELM–Adaboost model	5.003 s
		Experiment 2: KPLS + ZOA feature selection + ELM–Adaboost model	7.127 s
		Experiment 2: KPLS + GSA feature selection + Hybrid classifiers with soft voting	9.413 s
		Experiment 2: Kriging Model + PSO feature selection + ELM–Adaboost model	9.387 s
		Experiment 2: Kriging Model + hybrid GSA-PSO feature selection + ELM–Adaboost model	5.609 s

## Data Availability

A publicly available dataset was used to perform the experiment and the database can be found in “Mazzi C, Massironi G, Sanchez-Lopez J, De Togni L, Savazzi S (2020) Face recognition deficits in a patient with Alzheimer’s disease: amnesia or agnosia? The importance of electrophysiological markers for differential diagnosis. FrontAgingNeurosci12:580609” [61].
